# High Level of METTL7B Indicates Poor Prognosis of Patients and Is Related to Immunity in Glioma

**DOI:** 10.3389/fonc.2021.650534

**Published:** 2021-04-29

**Authors:** Yujia Xiong, Mingxuan Li, Jiwei Bai, Yutao Sheng, Yazhuo Zhang

**Affiliations:** ^1^ Beijing Neurosurgical Institute, Capital Medical University, Beijing, China; ^2^ Department of Neurosurgery, Beijing Tiantan Hospital, Capital Medical University, Beijing, China; ^3^ Beijing Institute for Brain Disorders Brain Tumor Center, Beijing, China; ^4^ China National Clinical Research Center for Neurological Diseases, Beijing, China

**Keywords:** glioma, METTL7B, immune, prognosis, CGGA TCGA

## Abstract

Glioma is the most common primary intracranial malignant tumor in adults. Although there have been many efforts on potential targeted therapy of glioma, the patient’s prognosis remains dismal. Methyltransferase Like 7B (METTL7B) has been found to affect the development of a variety of tumors. In this study, we collected RNA-seq data of glioma in CGGA and TCGA, analyzed them separately. Then, Kaplan-Meier survival analysis, univariate and multivariate Cox analysis, and receiver operating characteristic curve (ROC curve) analysis were used to evaluate the effect of METTL7B on prognosis. Gene Ontology (GO), Kyoto Encyclopedia of Genes and Genomes (KEGG), Gene Set Enrichment Analysis (GSEA) enrichment analyses were used to identify the function or pathway associated with METTL7B. Moreover, the ESTIMATE algorithm, Cibersort algorithm, Spearman correlation analysis, and TIMER database were used to explore the relationship between METTL7B and immunity. Finally, the role of METTL7B was explored in glioma cells. We found that METTL7B is highly expressed in glioma, and high expression of METTL7B in glioma is associated with poor prognosis. In addition, there were significant differences in immune scores and immune cell infiltration between the two groups with different expression levels of METTL7B. Moreover, METTL7B was also correlated with immune checkpoints. Knockdown of METTL7B revealed that METTL7B promoted the progression of glioma cells. The above results indicate that METTL7B affects the prognosis of patients and is related to tumor immunity, speculating that METTL7B may be a new immune-related target for the treatment of glioma.

## Introduction

Glioma is the most common primary malignant brain tumor in adults (1, 2). Glioma can be divided into low grade glioma (WHO grade II/III, LGG) and glioblastoma (WHO grade IV, GBM) (3). WHO 2016 classified glioma according to molecular pathological type (IDH mutation and 1p/19q Codeletion), which will be helpful for clinical treatment (4, 5). Currently, surgery, radiotherapy, and alkylating agent are the main treatment options for glioma (6–8). However, the prognosis of glioma as a whole is poor (9, 10). Recent studies find that tumor immune response plays an essential role in glioma (11–13), suggesting the promising prospect of immune therapy for glioma therapy.

METTL7B, located at chromosome 12q13.2, is correlated with methyltransferase activity and s-adenosine methionine-dependent methyltransferase activity. It has been found that METTL7B contributes to the occurrence and development of breast cancer, thyroid cancer, and lung cancer (14–16). Moreover, recent research revealed that METTL7B may regulate immunity by regulating the methylation of the FOX3P promoter (17), a novel immune-associated gene.

The current study analyzed the glioma transcriptome data in the TCGA and CGGA database and found that METTL7B is highly expressed in glioma and is correlated with multiple clinical features of glioma. Moreover, patients with high METTL7B levels have a poor prognosis. At the same time, we analyzed the differential genes between the two groups with high and low expression of METTL7B and performed enrichment analysis based on the differential genes, and the results indicated enrichment of several immune-related functions and pathways. The results showed that METTL7B is positively correlated with the ESTIMATE score. Also, we found that METTL7B is associated with multiple immune checkpoints, and the immune cell subpopulations may be associated with METTL7B. Moreover, knockdown of METTL7B decreased the proliferation, migration, and invasion ability of glioma cells. In summary, our findings revealed that METTL7B affects the prognosis of patients and is involved in tumor immunity in glioma.

## Materials and Methods

### Data Download and Collation

We downloaded the glioma RNA-seq data (total 698 cases, glioblastoma (GBM) 169 cases, low-grade glioma (LGG) 529 cases), and clinical data (1114 cases, GBM+LGG) from the TCGA website (http://www.tcga.org/). After deleting the missing samples of clinical data, gene expression and the corresponding clinical documents of 640 cases were obtained. RNA-seq and clinical data for glioma (325 + 693 cases) were downloaded from the CGGA website (http://www.cgga.org.cn). For batch correction and integration, the LIMMA (18) package and SVA (19) package were used.

### GEPIA Analysis and HPA Analysis

The GEPIA website was used to analyze the differences between the METTL7B gene expression levels in glioma (LGG and GBM) and normal samples. Moreover, we used the GEPIA website to draw Kaplan–Meier (20) curves for survival analysis in the TCGA database. We further verified the METTL7B protein levels of normal samples and glioma samples using the Human Protein Atlas (HPA website, http://www.proteinatlas.org).

### Prognostic Analysis

Kaplan-Meier curve was used for survival analysis. We used R software to load the survival package (https://CRAN.R-project.org/package=survival) and the survminer package (https://CRAN.R-project.org/package=survivalminer) to draw the Kaplan-Meier curve on glioma samples of the CGGA database. We also performed univariate and multivariate Cox analysis, and receiver operating characteristic curve (ROC) survival analysis was performed using the survivalROC package (https://CRAN.R-project.org/package=survivalROC).

### Differential Gene Enrichment Analysis

Differential analysis was performed using the LIMMA package. The clusterProfiler (21) package and enrichplot package (https://github.com/GuangchuangYu/enrichplot) was applied to perform the GO and KEGG enrichment analysis of differential genes. In addition, GSEA software was used to analyze the GO and KEGG pathways between the high and low levels of METTL7B.

### Immune Evaluation

The immune score of samples was assessed in the R software using the ESTIMATE package (https://R-Forge.R-project.org/projects/estimate/). Cibersort (22) algorithm was applied to analyze the correlations between METTL7B and 22 immune cell subsets. Tumor Immune Estimation Resource (TIMER, https://cistrome.shinyapps.io/timer/) was further used to analyze the relationship between different immune cells and prognosis in GBM and LGG and the correlations between METTL7B and immune cells.

### Cell Line and Transfection

U87 Glioma cell line, purchased from the American Type Culture Collection, was cultured in DMEM (Thermo Fisher Scientific) supplemented with 10% fetal bovine serum in an incubator at 37°C and 5% CO2. The METTL7B small interfering RNA (siRNA) and negative control (NC) were obtained from the Guangzhou RiboBio Co., Ltd. Cells were seeded in the 6-well plate (5×10^5^ cells per well)were transfected with siRNAs and NC with the help of lipofectamine 3000.

### Quantitative Reverse Transcription Polymerase Chain Reaction (qRT-PCR) and Western Blot

Two days after transfection, cells were collected for RNA using Trizol reagent (Invitrogen). The cDNA was further synthesized using the SuperScript III First Strand Synthesis System (Invitrogen). QuantStudio 5 (Applied Biosystems) was used to perform the qRT-PCR. The expression of METTL7B was normalized to GAPDH. The primers of MTEEL7B were as following: CCTGCCTAGACCCAAATCCC (forward) and AAACCGCTCATATTGGAGGTG (reverse). For western blot, mouse METTL7B antibody (Santa Cruz Biotechnology, sc-398626, 1:500) was used as primary antibody, and the β-actin was applied as the control.

### CCK8, Migration, and Invasion Assay

Glioma cells were cultured in the 96-well plate (5x10^3^ cells per well) for 24 hours before transfection. Cells were then treated with METTL7B siRNAs or NC, and the optical density (OD) values at 450nm at several time points were assessed after the 3h-incubation of 10ul CCK8 assay at 37°C. 1x10^5^ glioma cells transfected with METTL7B siRNAs and NC were seeded in the transwell chamber (Corning) with (For invasion) or without (For migration) Matrigel. After 4 hours (migration) and 8 hours (invasion), the cells at the bottom surface of the filters were fixed and stained with 0.1% crystal violet.

### Statistical Analysis

The R software (version 3.6.3) was used for the statistical analysis. The median value of METTL7B expression was considered as the cutoff value to separate patients into the high and low groups. The Circlize (23) and Corrplot packages (https://github.com/taiyun/corrplot) were used to map the correlation circles. Other R packages, “ggplot2”, “ggpubr”, “vioplot” were applied to visualize the results of data analysis. Wilcoxon Signed Rank test and Student’s t test were used for statistical analysis between two groups, while the Kruskal-Wallis test was applied for statistical tests of more than two groups. When p less than 0.05, we considered the difference to be statistically significant.

## Results

### METTL7B Is Highly Expressed in Glioma and Is Related to Patient Prognosis

**Figure 1:**
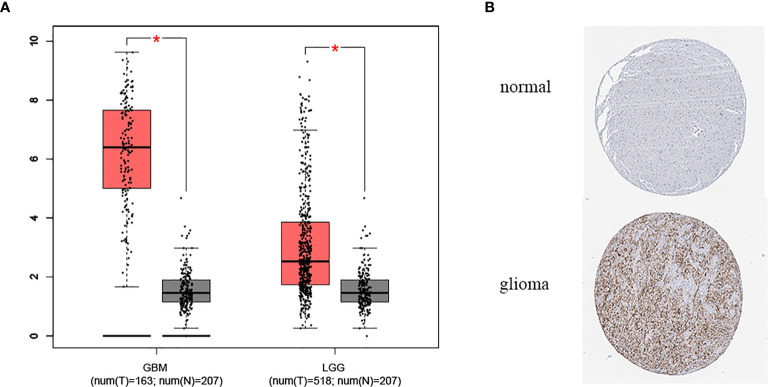
The expression level of METTL7B in glioma. **(A)** Expression of METTL7B in glioma and normal tissues in GEPIA database. **(B)** The protein level of METTL7B in glioma and normal tissues based on the Human Protein Atlas. *p < 0.05.

**Figure 2:**
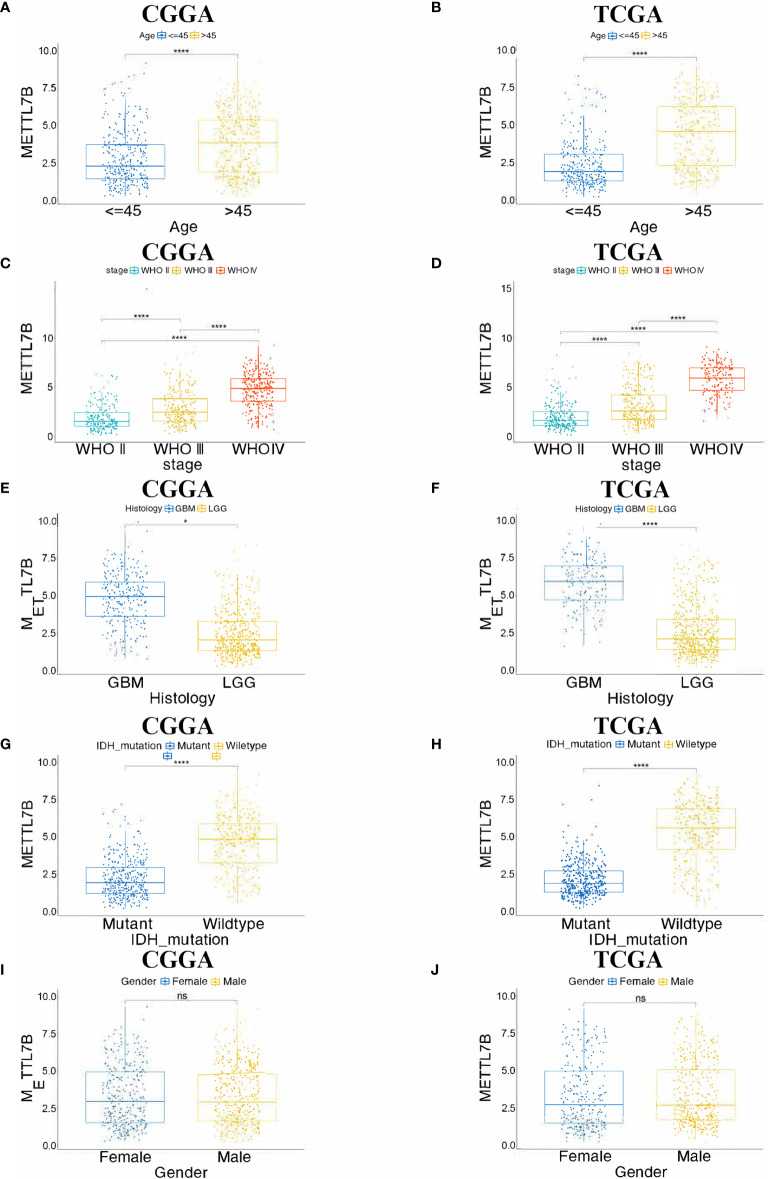
Correlation between the expression of METTL7B and clinical features using CGGA and TCGA database. **(A, B)** Differential expression of METTL7B was significantly related to the age of the patients, **(C, D)** WHO stage of glioma, **(E, F)** histology, **(G, H)** IDH_mutation. **(I, J)** The expression level of METTL7B was not correlated with the Gender of patients. *p < 0.05; ****p < 0.0001; ns, not significant.

The expression of METTL7B in tumor samples of glioma and normal samples was evaluated using the GEPIA website and we found that METTL7B is highly expressed in both LGG and GBM samples (**Figure 1A**). Similarly, the protein level of METTL7B in glioma samples was also higher compared to that in normal samples in the HPA database (**Figure 1B**). Next, we analyzed the relationships between METTL7B expression level and the clinicopathological characteristics of glioma patients, showing that the expression of METTL7B was significantly correlated with age, tumor stage, pathology, and IDH1mutation (*p* < 0.001), but not with gender or radiotherapy in glioma (*p* > 0.05) (**Figure 2**, **Table 1**, **Supplementary Figure 1**).

**Table 1:** Differences in clinical characteristics between the high and low METTL7B expression groups.

Database	Clinical features	P-value
TCGA	Histology	<0.001*
TCGA	Grade	<0.001*
TCGA	Gender	0.48
TCGA	Age	<0.001*
TCGA	IDH mutation	<0.001*
CGGA	PRS type	<0.001*
CGGA	Histology	<0.001*
CGGA	Grade	<0.001*
CGGA	Gender	0.64
CGGA	Age	<0.001*
CGGA	Radio status	0.63
CGGA	Chemo status	<0.001*
CGGA	IDH mutation status	<0.001*
CGGA	1p19q codeletion status	<0.001*

*indicated p < 0.05; PRS, primary and recurrent status.

**Figure 3:**
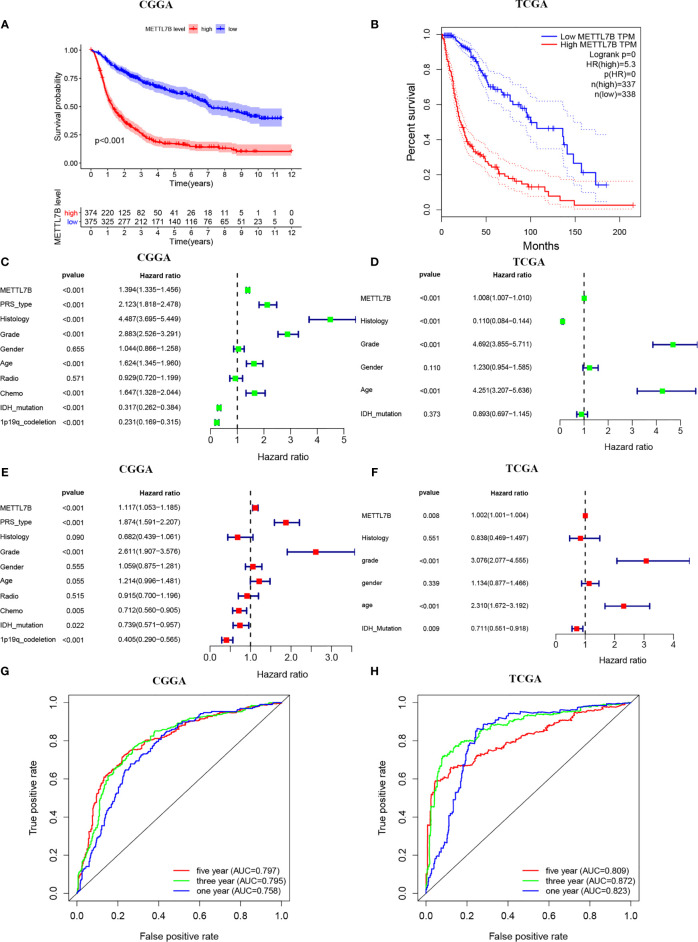
Survival analysis of METTL7B in CGGA and TCGA patients. **(A, B)** Kaplan-Meier survival curves in the high and low expressions of METTL7B groups. **(C, D)** Univariate Cox analysis of METTL7B. **(E, F)** Multivariate Cox analysis of METTL7B. **(G, H)** ROC analysis of METTL7B for 1, 3, and 5-year survival.

Kaplan-Meier survival analysis indicated that the high expression of METTL7B was significantly correlated with poor prognosis (*p <*0.001, **Figures 3A, B**). Furthermore, univariate Cox analysis identified METTL7B as a risk factor, and subsequent multivariate Cox analysis revealed that METTL7B is independently associated with the prognosis in glioma (**Figures 3C–F**). Moreover, we confirmed that PRS type, histology, grade, chemotherapy, IDH mutation, and 1p19q codeletion could also affect the prognosis of the patients (**Figures 3C–F**).

In addition, the ROC curve analysis suggested that METTL7B shows satisfactory performance in predicting the 1-year, 3-year, and 5-year survival rates of patients (all AUC>0.7) (**Figures 3G, H**).

### Differential Gene Enrichment Analysis Between METTL7B Groups

**Figure 4:**
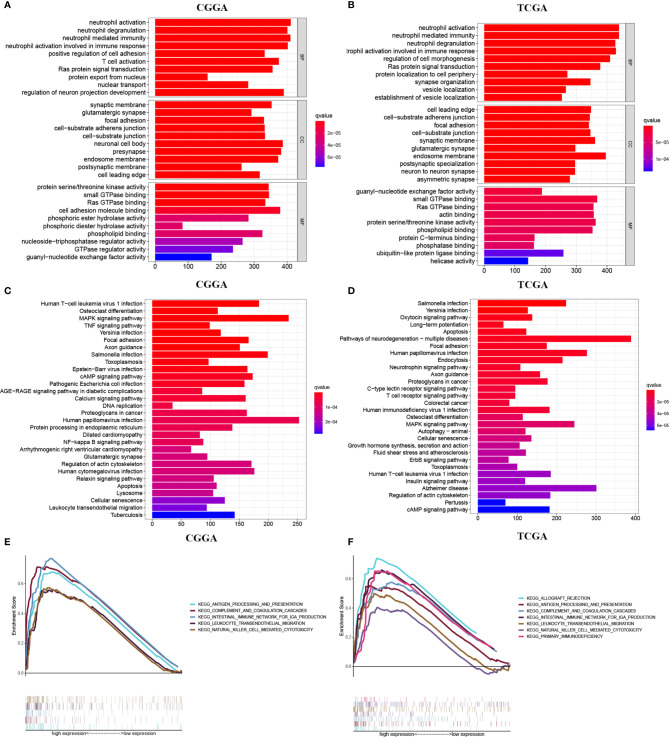
Differential gene enrichment analysis between different METTL7B groups. **(A, B)** Top 10 GO terms, involving BP, CC, and MF. **(C, D)** Top 30 KEGG pathways. **(E, F)** GSEA enrichment analysis revealed potential associations between METTL7B and several immune-associated pathways.

We then analyzed the different genes, and the heatmap was developed to show the top 100 up-regulated and the top 100 down-regulated differential genes between the two groups (**Supplementary Figure 2**, **Supplementary Table 1**). Further GO enrichment analysis of differential genes revealed that METTL7B may be associated with neutrophil-mediated immunity, neutrophil activation, neutrophil activation involved in immune response, neutrophil degranulation, T cell activation, and other immune-related functions (**Figures 4A, B**). Interestingly, KEGG analysis further suggested that METTL7B may be involved in some immune-related and previously recognized oncogenic pathways such as TNF signaling pathway, T cell receptor signaling pathway, NF-kappaB signaling pathway, MAPK signaling pathway, human T-cell leukemia virus type 1 infection, salmonella infection, and Yersinia infection, (**Figures 4C, D**). In addition, GSEA enrichment analysis in TCGA and CGGA databases also indicated significant enrichment of multiple immune-related functions and pathways (**Figures 4E, F**, **Supplementary Figure 3**). The above results indicated that METTL7B may function *via* the involvement of the tumor immune microenvironment. Thus, we further analyzed the relationship between METTL7B and tumor immunity.

### METTL7B Is Involved in Tumor Immunity in Glioma

**Figure 5:**
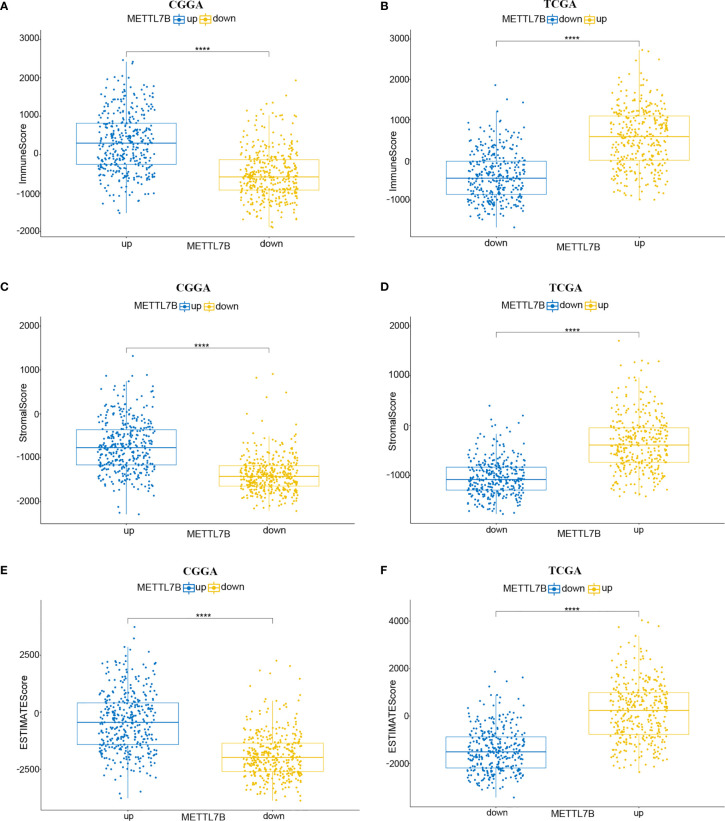
Relationship between ESTIMATE score and METTL7B expression level in CGGA and TCGA patients. **(A, B)** Immunescore, **(C, D)** stromalscore, and **(E, F)** ESTIMATEscore were higher in the group with higher METTL7B expression. ****p < 0.0001.

**Figure 6:**
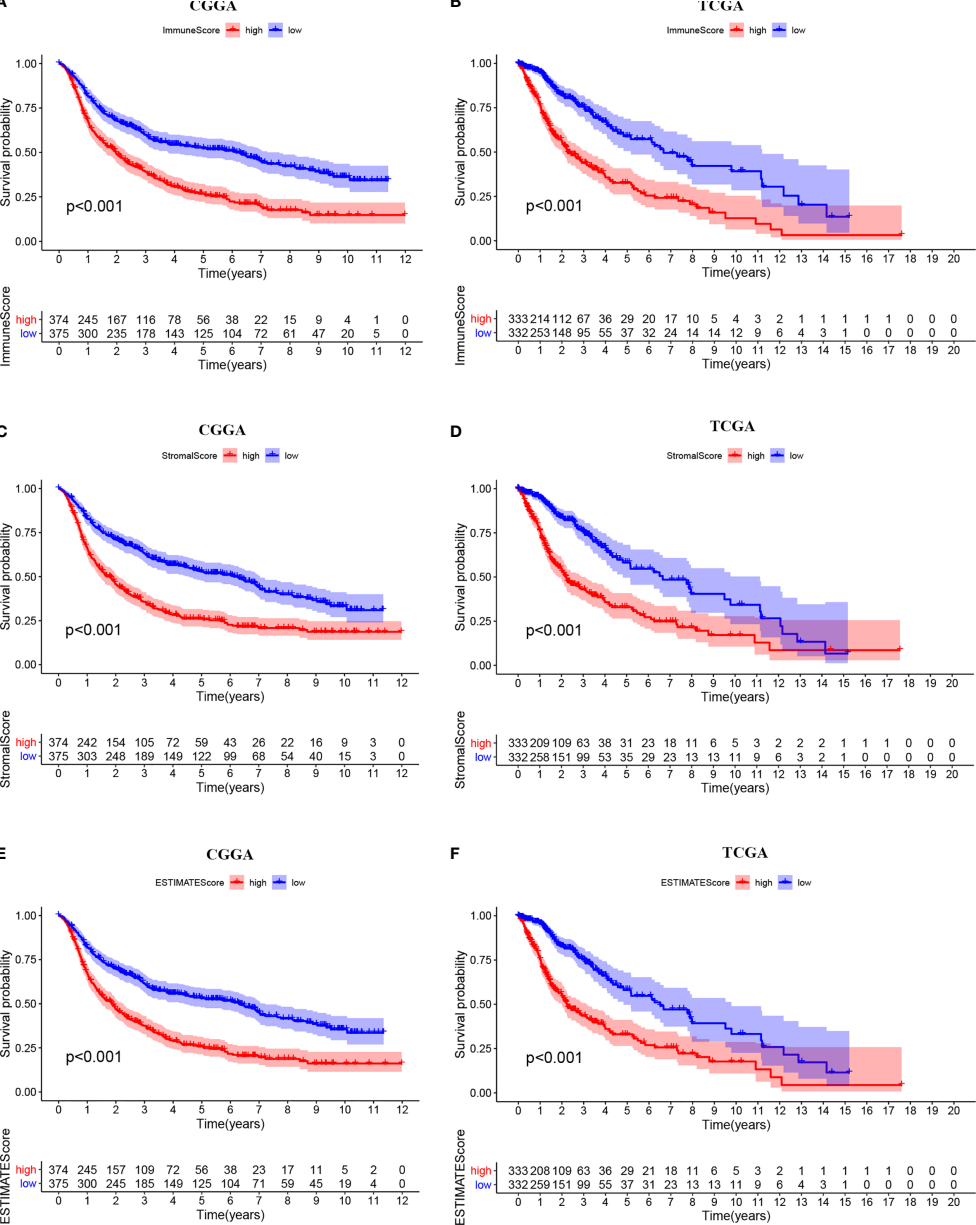
Kaplan-Meier survival curves of ESTIMATE score. High levels of **(A, B)** immunescore, **(C, D)** stromalscore, and **(E, F)** ESTIMATEscore correlated with poor prognosis.

**Figure 7:**
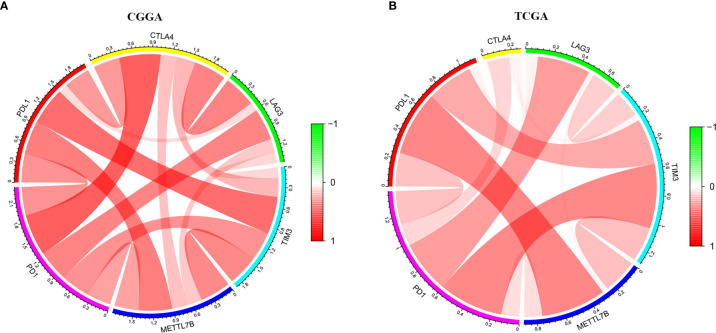
The circle diagram showed that METTL7B was positively correlated with multiple immune checkpoints, PD1, PDL1, CTLA4, LAG3, and TIM3 in glioma patients. **(A)** CGGA. **(B)** TCGA.

**Figure 8:**
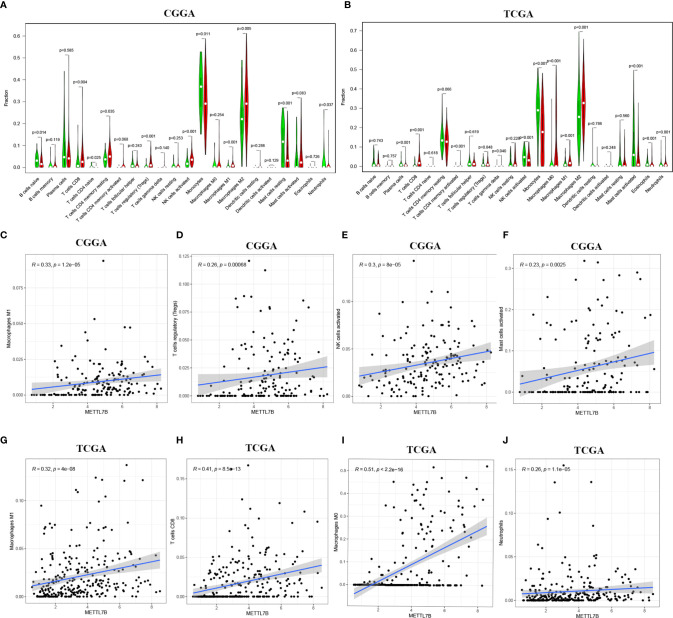
Proportions of the 22 types of tumor-infiltrate immune cells in different METTL7B groups in **(A)** CGGA and **(B)** TCGA. Correlation analysis between METTL7B and 22 kinds of immune cells in glioma. METTL7B was positively associated with **(C)** Macrophages M1, **(D)** T cells regulatory, **(E)** NK cells activated, **(F)** Mast cells activated in CGGA. **(G)** Macrophages M1, **(H)** T cells CD8, **(I)** Macrophages M0, **(J)** Neutrophils infiltration were positively correlated with METTL7B expression in TCGA.

**Figure 9:**
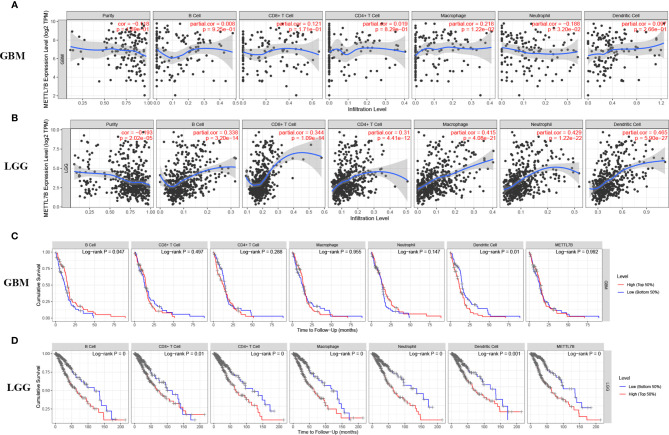
The relationship between METTL7B expression level, tumor purity, and immune cell infiltration was explored using the TIMER database. METTL7B was significantly correlated with immune cell infiltration in **(A)** GBM and **(B)** LGG patients. Kaplan-Meier survival analysis of several immune cells in **(C)** GBM and **(D)** LGG patients.

The ESTIMATE algorithm was performed to assess the immune levels of glioma patients, showing the significant differences (*p*<0.001) in the immune score, stromal score, and ESTIMATE score between the patients with high and low METTL7B expression. Specifically, the immune score, stromal score, and ESTIMATE score of the high expression METTL7B patients were all higher (**Figure 5**). Moreover, high immune score, high stromal score, and high ESTIMATE score were all associated with poor prognosis in glioma (**Figure 6**). Univariate cox analysis also confirmed the survival performance of the immune score, stromal score, and ESTIMATE score (**Supplementary Figure 4**). We further explored the correlations between METTL7B and immune checkpoints and identified that METTL7B is positively correlated with several immune checkpoints PD1, PDL1, CTLA4, LAG3, and TIM3 (**Figure 7**). Moreover, we analyzed the proportion of 22 immune cells in the two groups by the Cibersort algorithm, revealing that there were significant differences in T cells CD8, NK cells activated, Monocyte, Macrophages M1, Macrophages M2, and Neutrophils between the two groups with high and low METTL7B expression level (**Figures 8A, B**, **Supplementary Table 2**). The correlations between METTL7B and 22 kinds of immune cells were further analyzed by Spearman correlation analysis, and we found that METTL7B may correlate with multiple immune cells, including Neutrophils, MacrophagesM1, MacrophagesM2, T cells, and other immune cells (**Figures 8C–J**, **Supplementary Table 3**). Further analysis showed that compared with M1 chemokine, METTL7B was mainly related to M2 chemokine (**Supplementary Figure 5**). Finally, we used the TIMER database to explore these correlations in LGG or GBM patients alone. And the results suggested that in LGG patients, B cell, CD8+ T cell, CD4+ T cell, Macrophage, and Neutrophil significantly affect the prognosis (*p*<0.05), and were still correlated with METTL7B expression, while no correlations were found in GBM patients (**Figure 9**). In summary, the above findings showed that METTL7B is associated with the immune score, immune checkpoints, and immune cell infiltration in glioma patients.

### Inhibition of METTL7B Decreased the Proliferation, Migration, and Invasion Ability of Glioma Cells

**Figure 10:**
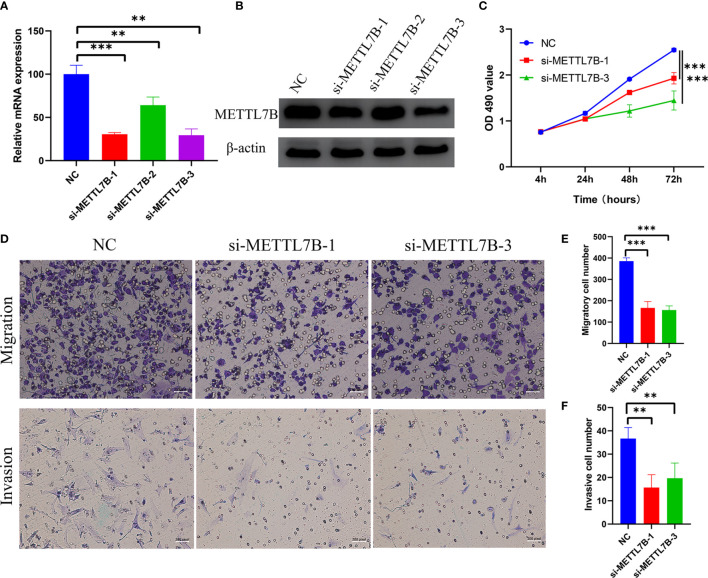
**(A)** qRT-PCR revealed that the METTL7B mRNA level was significantly suppressed using METTL7B siRNAs. **(B)** Western blotting indicated that the expression of METTL7B was decreased after treated with METTL7B siRNAs. **(C)** CCK8 assay showed that the proliferation ability of glioma cells was significantly decreased after knockdown of METTL7B. **(D–F)** Transwell assay revealed that knockdown of METTL7B inhibited the migration and invasion ability of glioma cells. Magnification, 200X. **p < 0.01; ***p < 0.001.

To further validate the potential oncogenic role of METTL7B in glioma, we explored its function in the glioma cell line. Cells were treated with three siRNAs targeting METTL7B (si-METTL7B-1, si-METTL7B-2, and si-METTL7B-3), and the qRT-PCR (**Figure 10A**) and western blot (**Figure 10B**) revealed that si-METTL7B-1 and si-METTL7B-3 effectively inhibited the expression of METTL7B. Therefore, the two siRNAs were selected for further experiments. Cell proliferation ability was significantly reduced after METTL7B knockdown (**Figure 10C**). Moreover, inhibition of METTL7B significantly decreased the cell migration and invasion ability (**Figures 10D–F**).

## Discussion

Glioma is the most common primary tumor of the central nervous system, accounting for 15% of all brain tumors (24). At present, the effect of targeted drug therapy is not satisfactory (13). Among glioma, glioblastomas are highly resistant to many chemotherapeutic drugs (25). Recent studies have shown that radiotherapy combined with chemotherapy can improve patient survival (26), however, the prognosis of patients is dismal (27). Exploring new therapeutic targets and targeted therapeutic drugs for glioma patients are of no delay.

In this study, Kaplan-Meier analysis, univariate and multivariate Cox analysis, and ROC curve were employed to analyze the relationships between METTL7B and the clinical characteristics and prognosis of patients. Moreover, GO, KEGG, and GSEA enrichment analysis was conducted to identify the potential mechanisms of METTL7B in glioma. Further analysis of immune infiltration was carried out to explore the relationship between the patient’s prognosis and immune cells. Importantly, the relationships between METTL7B and immune checkpoints were analyzed, and the differences of 22 kinds of immune cells in patients with high and low METTL7B levels were further analyzed. Finally, the role of METTL7B in glioma cells was explored.

METTL7B is associated with the development of a variety of tumors, while its role in glioma has not been previously studied. Through GEPIA analysis, we found that METTL7B was highly expressed in glioma (LGG and GBM), and the expression of METTL7B in GBM was increased compared to that of LGG. Similarly, HPA results also support the high expression of METTL7B in glioma. These results suggest that METTL7B may contribute to glioma progression. Further analysis identified that the expression of METTL7B was higher in glioma with a higher WHO grade and the METTL7B level in IDH1 wild-type was higher than that of mutant type. Accumulating studies have shown that IDH1 mutations are related to the occurrence and development of glioma (28), and IDH1 mutations are more common in LGG(WHO II, WHO III) than in GBM(WHO IV) (29). Therefore, we speculated that the higher expression of METTL7B in GBM patients might due to more GBM patients are IDH wild-type tumors, which expressed higher METTL7B levels.

The Kaplan-Meier curves of METTL7B indicated that METTL7B significantly affected the prognosis of glioma patients. To verify the role of METTL7B in the prognosis of glioma patients, univariate and multivariate Cox analyses were conducted, which suggested that METTL7B might independently predict the prognosis of glioma patients. ROC curve further verified our results, the analysis of CGGA and TCGA databases showed that the performance of METTL7B for 1-year, 3-year, and 5-year OS prediction was satisfactory.

To explore the possible mechanism of METTL7B affecting the survival of patients, GO and KEGG enrichment analyses were performed based on the differentially expressed genes between METTL7B groups, and the results suggested various immune-related pathways. In addition, GSEA enrichment analysis further revealed enrichment of multiple immune-related functions and pathways. Therefore, we assumed that METTL7B may function *via* regulation of tumor immunity, such as regulation of neutrophils, T cell activation, and B cell-mediated immunity, which was not revealed in previous studies.

Patients with higher expression of METTL7B had a higher ESTIMATE score, stromal score, and immune score, which were adverse prognosis factors, further confirming that METTL7B may participate in the tumor immune microenvironment. Moreover, METTL7B was positively correlated with multiple immune checkpoints. Studies have shown that the upregulation of immune checkpoints such as PD-L1, CTLA-4, TIM-3, and LAG3 in glioma helps tumor immune evasion, leading to T cell dysfunction (30–32), suggesting that METTL7B may promote tumor immune evasion by upregulating the expression of immune checkpoints. We also used the Cibersort to evaluate the ratio of different immune cells which found significant differences between the different METTL7B expression groups. Wherein, Neutrophils, Macrophages M1, Macrophages M2, and T cells correlated with the expression of METTL7B, which validated the immune-related findings of the enrichment of differentially expressed genes. Macrophages are the main immune cells in glioma and can be polarized into M1 and M2 macrophages under the influence of chemokines (33). M2 macrophages are an immunosuppressive phenotype in gliomas and are associated with the poor prognosis of patients (34). Our analysis found that METTL7B was mainly related to M2 chemokine, and thus we speculated that METTL7B might contribute to a tumor microenvironment favorable for tumor growth by promoting the differentiation of macrophages into M2 type. Collectively, our study found that the high expression of METTL7B was associated with poor prognosis in glioma patients, which may be mediated *via* inhibiting tumor immunity. Finally, the results of the TIMER database suggested that in LGG patients, several immune cells influenced patients prognosis and were associated with METTL7B expression, while these results were not consistent in GBM, suggesting the complex mechanisms of METTL7B in glioma and the latent function difference of METTL7B between LGG and GBM. Further mechanism exploration is highly warranted.

In the current study, glioma patients from TCGA and CGGA databases were simultaneously analyzed, and the consistent results of the two databases made our results more reliable. After analysis, we found a new glioma prognostic gene, METTL7B, which is also closely related to immunity. The result has not been reported in previous studies, so our study may influence the future diagnosis and treatment of glioma. However, our study also has several limitations. The function and potential mechanisms of METTL7B in glioma cells were not assessed in the current study. Moreover, the association between METTL7B and tumor immune microenvironment remains further validation. Finally, the role of METL7B in some special glioma types, such as diffuse midline glioma, remains further discussed.

## Conclusion

Our study revealed a novel prognostic gene, METTL7B, in glioma. Patients with high METTL7B expression have a poor prognosis and show a distinct immune landscape compared to those with low expression, identifying METTL7B as a promising target for drug and immune therapy in glioma.

## Data Availability Statement

Publicly available datasets were analyzed in this study. This data can be found here: http://www.tcga.org, http://www.cgga.org.cn.

## Ethics Statement

Ethical review and approval was not required for the study on human participants in accordance with the local legislation and institutional requirements. The patients/participants provided their written informed consent to participate in this study.

## Author Contributions

YX and YZ designed the study. YX wrote the manuscript and performed bioinformatics analysis. ML, JB, and YS contributed to manuscript discussion, figures and tables. All authors contributed to the article and approved the submitted version.

## Funding

This study was supported by the National Natural Science Foundation of China (81771489); supported by Beijing Municipal Science & Technology Commission (Z171100000117002).

## Conflict of Interest

The authors declare that the research was conducted in the absence of any commercial or financial relationships that could be construed as a potential conflict of interest.
