# Supplementary figures and images for: High Level of METTL7B Indicates Poor Prognosis of Patients and Is Related to Immunity in Glioma
Source: Front Oncol. 2021 Apr 29;11:650534. doi: 10.3389/fonc.2021.650534 (PMC8117938; doi:10.3389/fonc.2021.650534)

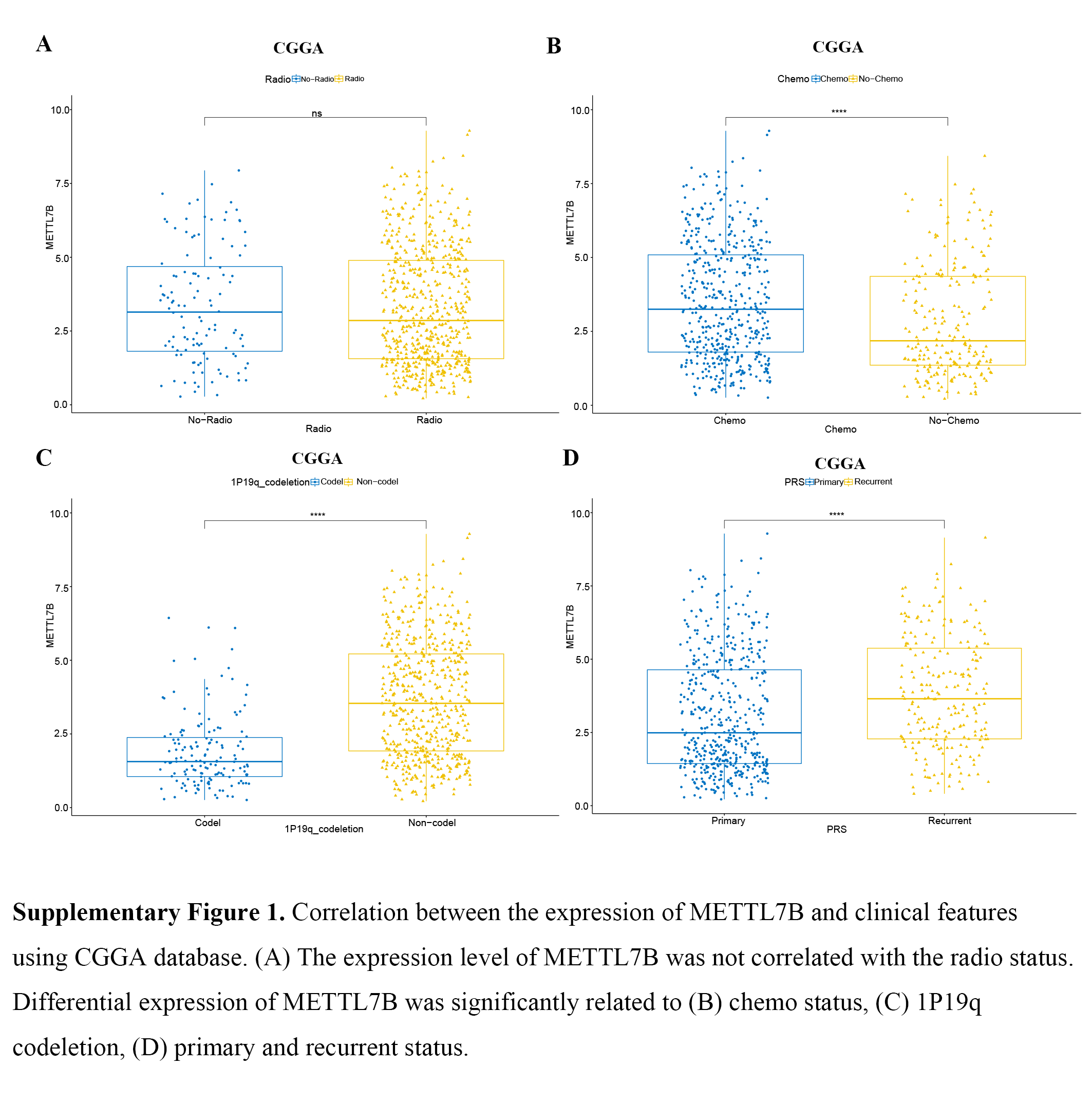

Supplement: Supplementary file 1 [file Image_1.tif]

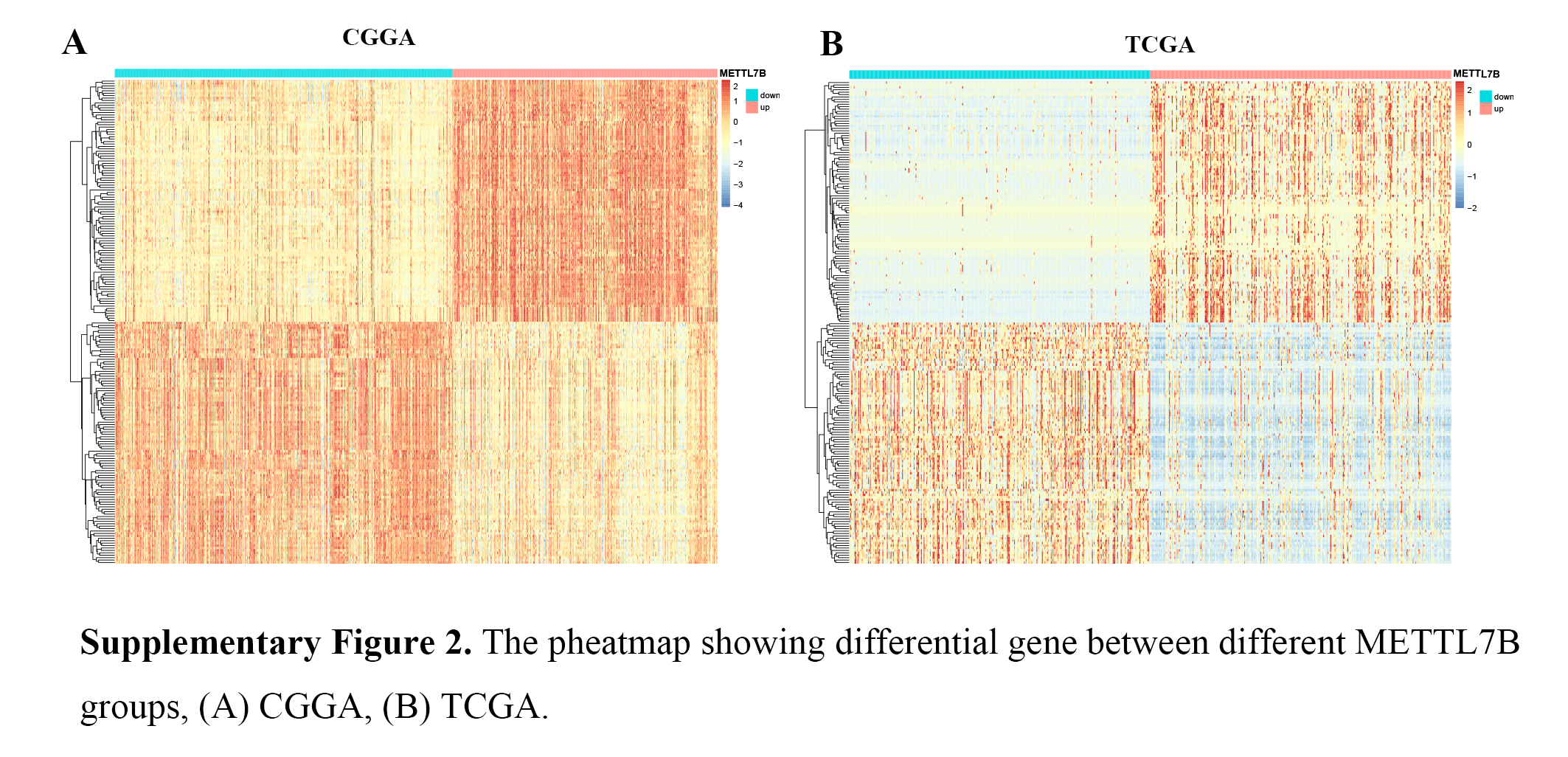

Supplement: Supplementary file 2 [file Image_2.tif]

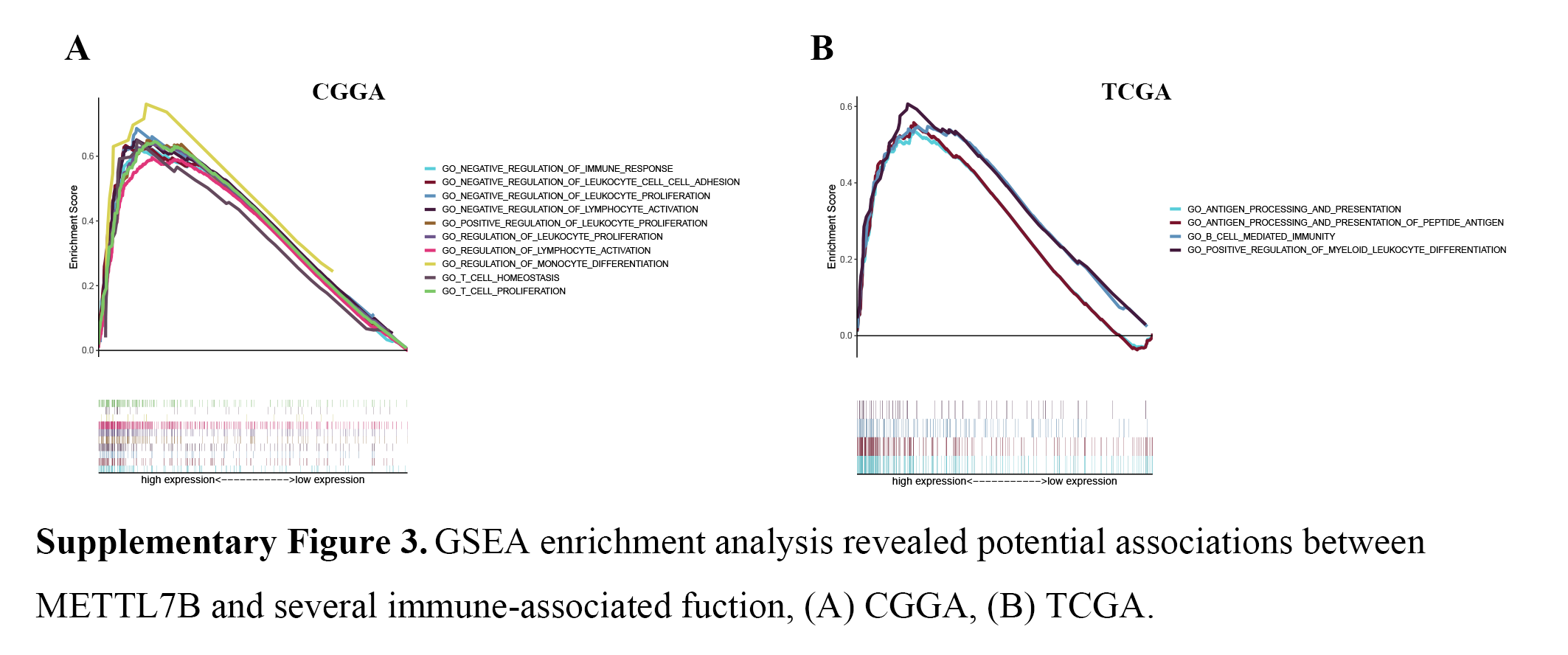

Supplement: Supplementary file 3 [file Image_3.tif]

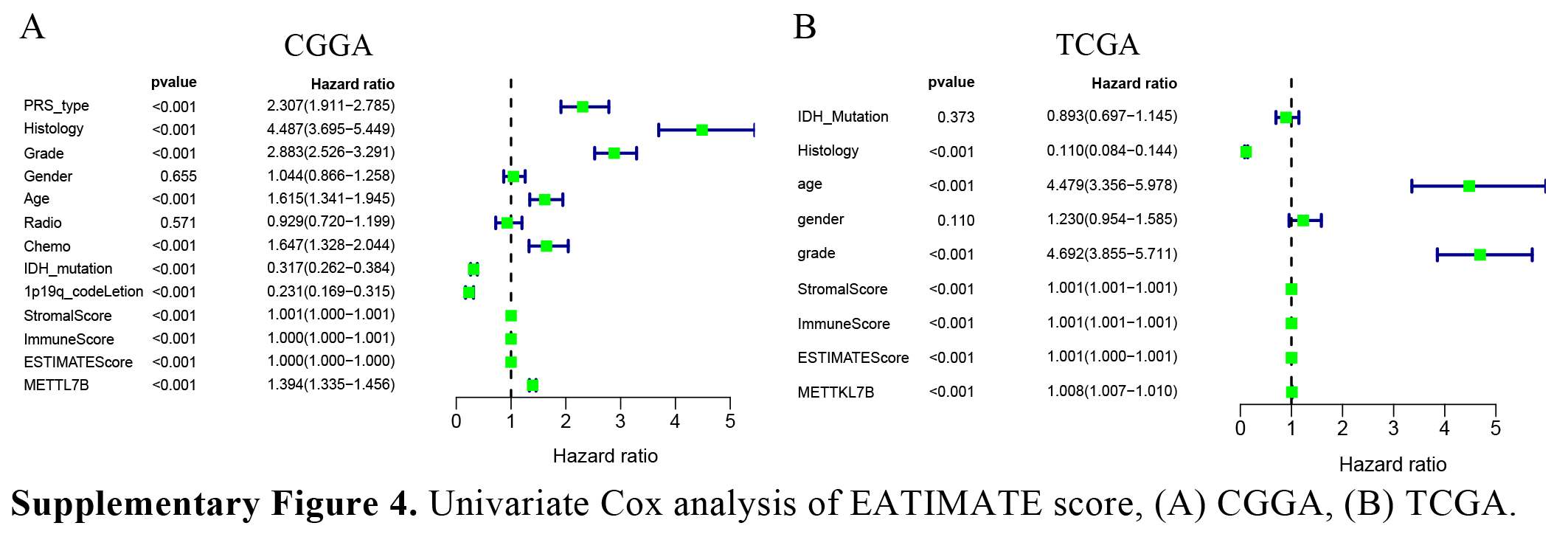

Supplement: Supplementary file 4 [file Image_4.tif]

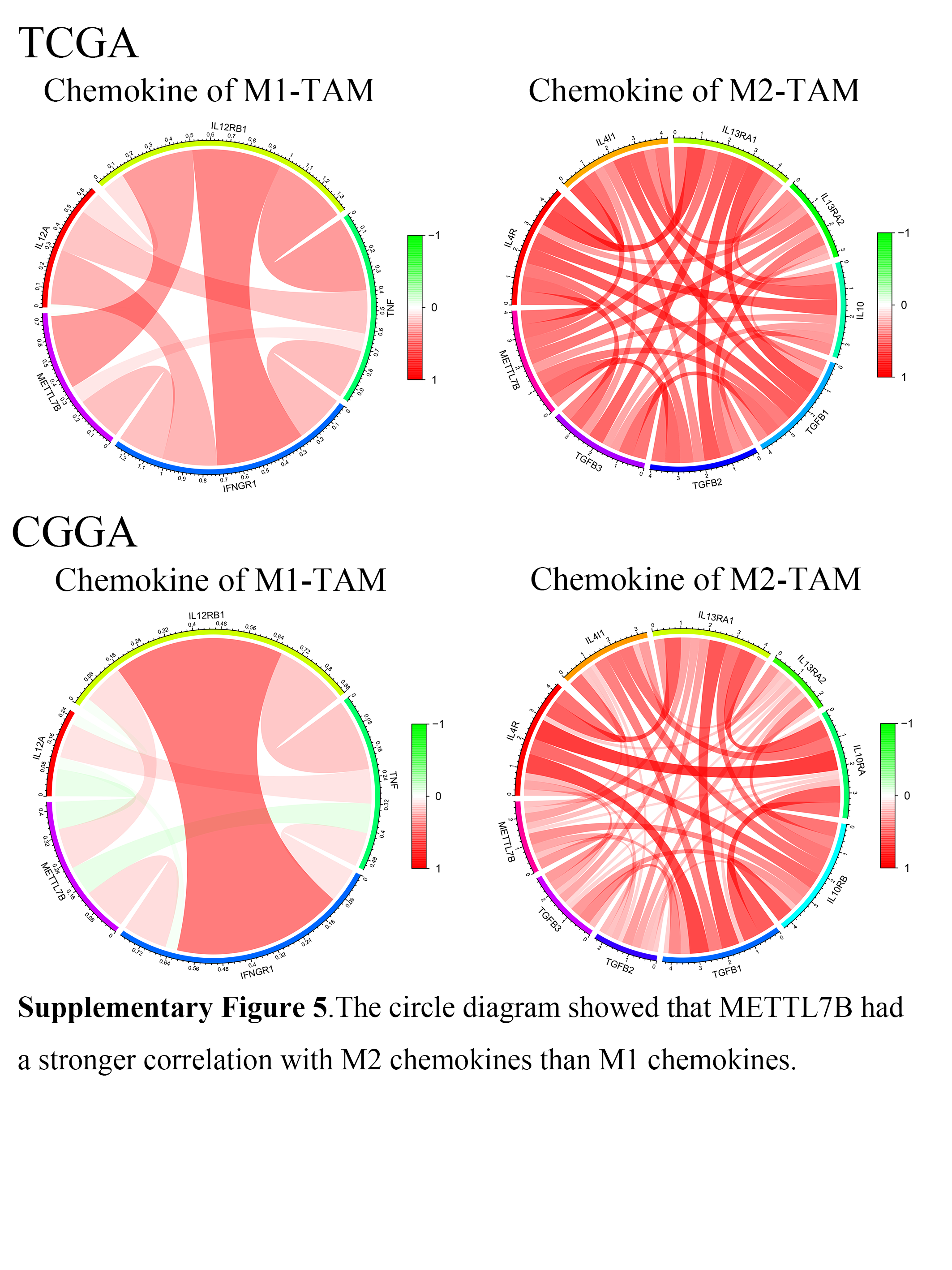

Supplement: Supplementary file 5 [file Image_5.tif]
