# Supplementary material for: High Level of METTL7B Indicates Poor Prognosis of Patients and Is Related to Immunity in Glioma
Source: Front Oncol. 2021 Apr 29;11:650534. doi: 10.3389/fonc.2021.650534 (PMC8117938; doi:10.3389/fonc.2021.650534)
Supplement: Supplementary file 7 [file Table_2.docx]

**Supplementary Table 2** Immune cells which have difference between the high and low METTL7B expression groups.

| **Database** | **Immune cells** | **P-value** |
| --- | --- | --- |
| TCGA | Plasma cells | <0.001 |
| TCGA | T cells CD8 | <0.001 |
| TCGA | T cells CD4 memory activated | <0.001 |
| TCGA | T cells gamma delta | 0.040 |
| TCGA | NK cells activated | <0.001 |
| TCGA | Monocytes | <0.001 |
| TCGA | Macrophages M0 | <0.001 |
| TCGA | Macrophages M1 | <0.001 |
| TCGA | Macrophages M2 | <0.001 |
| TCGA | Mast cells activated | <0.001 |
| TCGA | Eosinophils | <0.001 |
| TCGA | Neutrophils | <0.001 |
| CGGA | B cells naive | 0.014 |
| CGGA | T cells CD8 | 0.004 |
| CGGA | T cells CD4 naive | 0.025 |
| CGGA | T cells CD4 memory resting | 0.035 |
| CGGA | T cells regulatory (Tregs) | <0.001 |
| CGGA | NK cells activated | <0.001 |
| CGGA | Monocytes | 0.011 |
| CGGA | Macrophages M1 | <0.001 |
| CGGA | Macrophages M2 | 0.005 |
| CGGA | Mast cells resting | 0.001 |
| CGGA | Neutrophils | 0.037 |
