# Supplementary material for: High Level of METTL7B Indicates Poor Prognosis of Patients and Is Related to Immunity in Glioma
Source: Front Oncol. 2021 Apr 29;11:650534. doi: 10.3389/fonc.2021.650534 (PMC8117938; doi:10.3389/fonc.2021.650534)
Supplement: Supplementary file 8 [file Table_3.docx]

**Supplementary Table 3** Immune cells whose correlation P value is less than 0.05

| **Database** | **Cell** | **P-Value** | **R** |
| --- | --- | --- | --- |
| TCGA | Plasma cells | 0.0006 | -0.2 |
| TCGA | T cells CD8 | 8.49E-13 | 0.41 |
| TCGA | T cells CD4 memory activated | 9.31E-06 | 0.26 |
| TCGA | T cells gamma delta | 0.0019 | 0.18 |
| TCGA | NK cells resting | 0.0205 | 0.14 |
| TCGA | NK cells activated | 1.02E-08 | -0.33 |
| TCGA | Monocytes | 4.21E-20 | -0.51 |
| TCGA | Macrophages M0 | 8.75E-20 | 0.51 |
| TCGA | Macrophages M1 | 3.96E-08 | 0.32 |
| TCGA | Macrophages M2 | 4.42E-08 | 0.32 |
| TCGA | Dendritic cells activated | 0.0382 | -0.12 |
| TCGA | Mast cells activated | 1.89E-05 | -0.25 |
| TCGA | Eosinophils | 4.90E-08 | -0.32 |
| TCGA | Neutrophils | 1.10E-05 | 0.26 |
| CGGA | B cells naive | 0.0015 | -0.25 |
| CGGA | T cells regulatory (Tregs) | 0.0008 | 0.26 |
| CGGA | T cells gamma delta | 0.0247 | 0.17 |
| CGGA | NK cells activated | 0.0001 | 0.29 |
| CGGA | Macrophages M1 | 2.94E-05 | 0.32 |
| CGGA | Macrophages M2 | 0.0177 | 0.18 |
| CGGA | Mast cells resting | 0.0001 | -0.29 |
| CGGA | Mast cells activated | 0.0034 | 0.23 |
